# Blockade of CCR4 breaks immune tolerance in chronic hepatitis B patients by modulating regulatory pathways

**DOI:** 10.1186/s12967-023-04104-8

**Published:** 2023-04-21

**Authors:** Arshi Khanam, Alip Ghosh, Joel V. Chua, Shyam Kottilil

**Affiliations:** Division of Clinical Care and Research, Institute of Human Virology, University of Maryland School of Medicine, Baltimore, MD USA

**Keywords:** CHB, CCR4, CD4 and CD8 T cells, Tregs

## Abstract

**Background:**

Immunotargets including checkpoint inhibitors and toll-like receptor 8 agonists have recently gained attention for the recovery of hepatitis B virus (HBV)-specific T cell exhaustion in chronic hepatitis B(CHB). Chemokine receptors have a similar significant role during viral infections; however, their role in CHB remains poorly understood. Therefore, in this study we evaluated the role of chemokine receptor 4 (CCR4) in deriving immunosuppression during CHB.

**Methods:**

We characterized CCR4+CD8+ T cells in CHB and identified their involvement in immunosuppression. Further, we examined if CCR4 blockade with mogamulizumab antibody can recover the functional exhaustion in HBsAg-specific T cells.

**Results:**

CHB patients exhibit higher frequency of CCR4+CD8+ T cells that increase with higher HBsAg levels and fibrosis scores. In vitro, HBs antigen triggers CCR4 expression. These cells express multiple inhibitory receptors and exhibit immunosuppressive functions by producing excessive immunoregulatory cytokines IL-4, IL-5, IL-10 and TGF-β1. CCR4 Blockade significantly boosted HBsAg-specific antiviral-cytokine production(IFN-γ, TNF-α and IL-21) in T cells through enhancing their proliferation capacity and polarizing these cells towards T helper 1(Th1) and T follicular helper cells(T_FH_) in case of CD4 cells, and cytotoxic T cell 1(TC1) and cytotoxic T follicular(T_CF_) cells in case of CD8. Cytotoxic potential was improved, while no induction of immunosuppressive-cytokines was seen after anti-CCR4 treatment thereby eliminating the risk of treatment-induced immunosuppression. CCR4 blockade inhibited the development and effector function of Tregs by controlling their expansion and TGF-β1 production preventing Tregs-induced immunotolearance.

**Conclusions:**

CCR4 blockade reconstitutes antiviral immune response in T cells and limits the immunosuppressive functions of Tregs, representing them as a promising immunotherapeutic target for functional cure of CHB.

**Supplementary Information:**

The online version contains supplementary material available at 10.1186/s12967-023-04104-8.

## Background

The effective therapies for chronic hepatitis B (CHB) infection mainly rely on the nucleos(t)ide analogue that competently suppress virus replication and improve hepatic inflammation but are unable to efficiently eliminate the virus from the liver [1–6]. Also, CHB infected patients most often require long-term treatment that is associated with a risk of drug resistance, adverse reactions, nonadherence, and high cost [7]. Besides, discontinuation of the treatment imposes the risk of hepatitis B virus (HBV) reactivation [8]. Hence, there is a critical need for new therapeutic strategies to achieve functional cure in a short period of time avoiding complications associated with long-term use of antiviral therapies.

One of the main obstacles to HBV clearance is a defective adaptive immune response in CHB patients. These patients exhibit dysfunctional HBV-specific T cell response that is mainly attributed to the enrichment of co-inhibitory molecules, reduction in antiviral cytokines and cytotoxic functions and expansion of T regulatory cells (Tregs), which associate with viral persistence and further disease progression into fibrosis, cirrhosis, and hepatocellular carcinoma (HCC) [9, 10]. Impaired HBV-specific T cell response also influences humoral immunity by limiting the secretion of neutralizing antibodies by B cells and thus preventing viral elimination. Several studies and clinical trials are investigating immunotherapeutic approaches to boost up the antiviral immune response by either checkpoint blockade of inhibitory pathways such as programmed death-1 (PD-1) or using toll-like receptor 8 (TLR8) agonists. A clinical trial investigated the effect of PD-1 checkpoint blockade in virally suppressed hepatitis B e antigen (HBeAg) negative CHB patients using PD-1 inhibitor nivolumab and reported a significant increase in HBsAg-specific T cells in the circulation and a modest decline in hepatitis B surface antigen (HBsAg) level in most of the patients [11]. PD-1 blockade partially recovers dysfunctional virus-specific B cells when used in combination with B-cell maturing cytokines such as IL-2 and IL-21, suggesting PD-1 as a critical target for the retrieval of adaptive immune response. More recently, activation of TLR8 using an agonist is getting much attention owing to its capacity to induce host immunity via the stimulation of pro-inflammatory and immunomodulatory cytokines. TLR8 agonist, specifically GS-9688 (Selgantolimod) induces cytokine production in peripheral blood mononuclear cells and initiates the effector function by several immune cells, which highlights the potential of immunotherapeutic targets to control CHB infection [12, 13]. While the current research is focusing on the inhibitory receptors and TLRs as treatment options for CHB patients, the role of the chemokine receptors (CCRs) has been overlooked.

The primary function of the chemokine receptors superfamily is to mediate cell migration and homing to the site of infection and injury. However, emerging data suggest that these receptors perform several key regulatory functions [14, 15]. CCRs are cytokine/chemokine receptors present on the surface of the various immune cells that interact with different cytokines and chemokines. A single CCR could have a single or multiple ligands to interact with. Our previous data demonstrated that CCRs including CXCR1 and CXCR2 induce inflammation and hepatocyte death in HBV-related acute-on-chronic liver failure patients and blockade of these receptors by SCH 527,123 antagonist significantly inhibited cell death and inflammation by limiting the production of pro-inflammatory cytokines and reactive oxygen species (ROS) which signifies the potential role of CCRs during liver diseases [16]. The chemokine receptor 4 (CCR4) is expressed on circulating and tissue-resident T cells. Among the various T cell subsets, CCR4 is predominantly expressed by T helper type 2 (Th2) and T regulatory cells (Tregs). CCR4 interacts with CCL17 and CCL22, performs various functions including immune cells trafficking under inflammatory conditions, and may have a profound regulatory role when appropriately triggered by its ligands. Role of CCR4 is being extensively studied in different types of cancers including HCC where it induces cell migration and promotes tumor growth and metastasis [17]. Not only CCR4 but also its ligands CCL17 and CCL22 were found to be highly overexpressed in human and mouse liver cancers [18]. During HCC, malignant cells produce CCL22 and recruit CCR4+ Tregs which facilitate the immune escape of malignant cells [19]. Since CCR4 is mainly expressed on Th2 cells and Tregs, which are responsible for immunosuppressive microenvironment, anti-CCR4 antibodies such as mogamulizumab are being used to enhance anti-cancer immunity [20]. Considering its possible clinical application in HCC, CCR4 is receiving increasing attention recently as a potential therapeutic target. However, whether CCR4 expression is directly upregulated during the course of HCC and participates in the worsening of the condition, or it plays a role during earlier forms of the disease such as chronic HBV infection is not yet known. Therefore, in this study we investigated the role of CCR4 in patients with chronic HBV infection and tried to understand if CCR4 is involved in the suppression of HBV-specific T cell response. Further, we identified if blockade of CCR4 could reverse the immunosuppression of HBV-specific T cells, required for viral clearance. Here, we establish that CCR4+ CD8+ T cells maintain an immunosuppressive environment during CHB infection by producing immunoregulatory cytokines and limiting the production of antiviral cytokines. Blockade of CCR4 with mogamulizumab (anti-CCR4 antibody) monoclonal antibody markedly improved the production of antiviral cytokines including interferon-γ (IFN-γ), tumor necrosis factor-α (TNF- α) and interleukin-21 (IL-21) by CD4+ as well as CD8+ T cells by transitioning these cells towards T helper type 1(Th1) and T follicular helper cells (T_FH_) in case of CD4, while cytotoxic T cell type 1 (TC1) and cytotoxic T follicular (T_CF_) cells in case of CD8 T cells. Moreover, the frequencies and functions of T regulatory cells (Tregs), which inhibits the effector T cell response and participates in the immunosuppression during CHB, were declined post CCR4 blockade. Therefore, CCR4 might represent a potential therapeutic target to boost HBV-specific immunity in CHB patients, required for viral clearance.

## Patients and methods

### Study participants and samples

Peripheral blood samples were obtained from a total of 46 CHB patients and 32 HBV-vaccinated healthy controls (Vacc-HC) in heparinized and non-heparinized tubes after the written informed consent. All healthy subjects completed a standard course of HBV vaccination and were positive for anti-HBs antibodies. CHB patients were HBsAg positive for greater than 6 months and were positive for anti-HBc antibody, and presented clinical, biochemical, and virological evidence of chronic HBV infection. The exclusion criteria for CHB patients includes coinfection with hepatitis A virus (HAV), HCV, HDV, HEV and HIV. Patients who had concomitant autoimmune liver disease or any other known liver comorbidities were excluded in this study. The study protocol was approved by the Institutional Review Board at the University of Maryland, Baltimore, USA, and the study was conducted in compliance with the Declaration of Helsinki. Table 1 summarizes the demographic, clinical and virological parameters of the study subjects. Serum samples were collected from the non-heparinized blood tubes while plasma was separated after centrifugation of heparinized blood samples at 4000 rpm for 5 min. Samples were stored at – 80 °C until further use. Peripheral blood mononuclear cells (PBMCs) were isolated from blood by Ficoll-Hypaque density gradient centrifugation (GE Healthcare, Chicago, IL) and were used directly or cryopreserved. Cryopreservation was done in fetal bovine serum (FBS) with 10% dimethyl sulfoxide and samples were stored at − 140 °C liquid nitrogen until use.

**Table 1:** Demographic and clinical parameters of the study subjects

Characteristics	Vaccinated Healthy Controls (Vacc-HC) (n = 32)	Chronic Hepatitis B (CHB) (n = 46)	P value
Age (years)	40 (23–64)	45 (27–73)	NS
Gender			
Male	20	33	
Female	12	13	NS
ALT (U/L)	22 (18–33)	61 (11–193)	0.002
AST (U/L)	24 (19–31)	45(17–146)	0.005
Anti-HBs positive (n)	32	None	NA
HBeAg positive (n)	NA	9	NA
HBeAb positive (n)	NA	22	NA
HBeAb negative (n)	NA	14	NA
Unknown	NA	10	NA
HBsAg (IU/ml)	NA	3.9 × 10^4^(8.6–1.9 × 10^5^)	NA
HBV DNA (copies/ml)	NA	5.32 × 10^5^ (< 10–1.7 × 10^8^)	NA
Liver fibrosis	NA	F0–F2 (n = 30)	NA
		F3–F4 (n = 6)	NA
		Unknown (n = 10)	NA
HBV genotypes	NA	A (n = 11)	NA
		B(n = 5)	
		C(= 6)	
		D(1)	
		E (3)	
		Unknown (n = 20)	
Race (n)			NS
Asians	12	13	
African Americans	14	27	
White	6	5	
Caucasian	0	1	

Values have been displayed as median with range and number

*ALT* alanine aminotransferase, *AST* aspartate aminotransferase, *NA* not applicable, *NS* non-significant

### Virological parameters

Upon recruitment to the study, HBsAg and HBV DNA levels were tested. Serum HBsAg quantification was performed using highly sensitive HBsAg chemiluminescent immunoassay (CLIA) kit (AutoBio Diagnostic CO. Ltd) according to the previously described procedure mentioned elsewhere [21]. Detection of chemiluminescence was done by Synergy H1 plate reader (BioTek Instruments, Inc.). For HBV DNA quantification, serum samples were sent out to a commercial lab where HBV DNA was quantified by Real-time PCR.

For further details of material and methods, please refer to Additional file 1: Material S1.

## Results

### CHB patients exhibit higher CCR4 expression on T cells that increase with HBsAg levels and fibrosis score

**Fig. 1:**
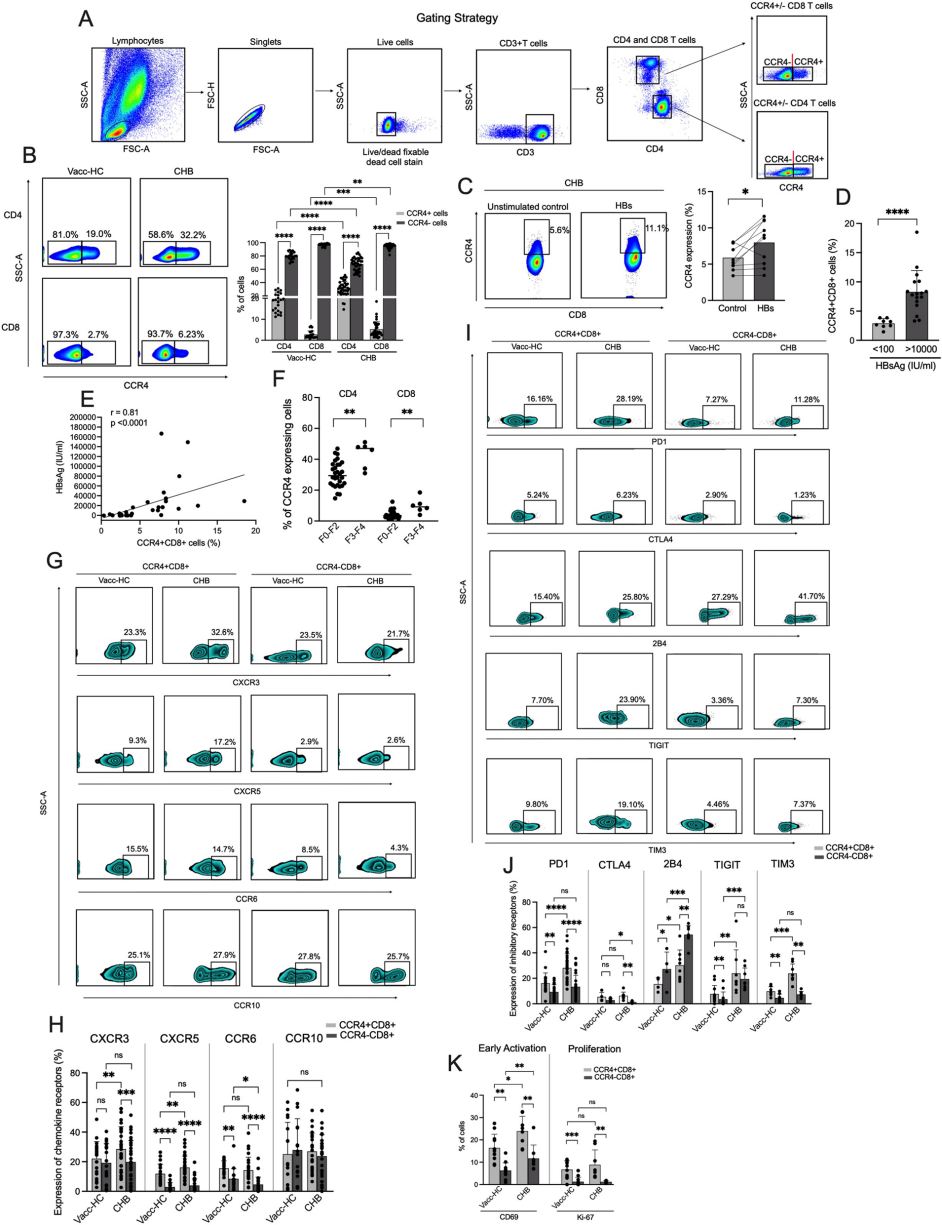
CHB Patients display higher expression of CCR4 on circulating T cells. **A** Gating strategy applied to analyze CCR4 expression on CD4 and CD8 T cells in the peripheral blood mononuclear cells (PBMCs) **B** Flow cytometry Pseudo color plots and cumulative data in the scatter plots represents the percent frequencies of CCR4+ and CCR4− CD4 and CD8 T cells in the PBMCs of chronic hepatitis B (CHB) patients (n = 37) and vaccinated-healthy controls (Vacc-HC) (n = 22) under ex vivo conditions. **C** Representative flow cytometry plots and accumulative data in line graph present the expression of CCR4 in CHB patients with and without HBs overlapping peptide stimulation (n = 10). **D** Frequencies of CCR4+CD8+ T cells in CHB patients with low (< 100) and high (> 10,000) HBsAg levels. **E** Correlation between HBsAg levels and CCR4+CD8+ T cells. **F** Frequencies of CCR4 expressing CD4 and CD8 T cells in CHB patients with different fibrosis scores (F0–F2) and (F3–F4). **G**, **H** Representative Zebra plot (**G**) and pooled data in the graphs (**H**) illustrate the expression of different chemokine receptors including CXCR3, CXCR5, CCR6 and CCR10 on CCR4 + CD8 + T cells in the PBMCs of CHB patients (PBMCs) (n = 32) and its comparison with Vacc-HC (n = 20) and CCR4-CD8 + counterpart. **I**, **J** Illustrative plots (**I**) and summary data in graphs (**J**) collected from the PBMCs of CHB patients and Vacc-HC indicate the expression of multiple inhibitory receptors PD1, CTLA4, 2B4, TIGIT and TIM3 on CCR4+CD8+ T cells and its comparison with CCR4-CD8 + counterpart. **K** Ex vivo analysis of early activation and proliferation of CCR4+CD8+ T cells by examining CD69 and Ki67 expression respectively. All the data in the graphs has been displayed as median with range. P value < 0.05 was considered statistically significant and defined as follows: *p < 0.05, **p < 0.01, ***p < 0.001 and ****p < 0.0001

Chemokine receptor 4 is primarily expressed by T helper 2 (Th2) type cells which induce immunosuppression by negatively regulating the immune response. We examined the expression of CCR4 on different subsets of T cells including CD4 and CD8 in chronic hepatitis B (CHB) patients and compared it with HBV-vaccinated healthy controls (Vacc-HC). To analyze the expression of CCR4 on CD4+ and CD8+ T cells, gating strategy has been illustrated in Fig. 1A. Our ex vivo data demonstrated higher expression of CCR4 on both CD4+ and CD8+ T cells in CHB patients than those of Vacc-HC (Fig. 1B). In addition, we compared the frequencies of CCR4- CD4 and CD8 T cells in CHB and Vacc-HC and found that the Vacc-HC exhibited a higher percentage of CCR4- CD4 and CD8 T cells in comparison to CHB. Since CCR4 expressing CD4 cells have already been studied, we aimed to characterize the phenotype and functions of CCR4 expressing CD8 T cells (CCR4+ CD8+) in CHB patients. We first examined whether HBV has any direct role in inducing CCR4 expression on CD8 T cells. For that reason, we stimulated CHB patients’ PBMCs with HBs overlapping peptides for 5 days and analyzed the change in CCR4 expression in CD8 T cells. We found a significant enhancement of CCR4 expression in CD8 T cells in most of the patient samples against HBs peptides, which indicates that HBV can directly trigger CCR4 expression (Fig. 1C). To confirm, if CCR4 expression is modulated by HBsAg levels, we segregated the CHB patients as per their HBsAg levels (low < 100 and high > 10,000) and checked the frequencies of CCR4+ CD8+ T cells. As expected, these cells were significantly higher in those with higher HBsAg levels (Fig. 1D). Moreover, correlation analysis further revealed strong association between HBsAg levels and CCR4 expression (Fig. 1E) which specifies the contribution of HBsAg in inducing CCR4 expression in CHB patients. In addition, we investigated if other HBV viral antigens such as hepatitis B core antigen (HBcAg) could induce CCR4 expression under in vitro setting and we observe an increase in CCR4 expression in few of the patients upon HBc peptide stimulation (Additional file 1: Fig. S1). As CCR4+ CD4+ T cells has been shown to be involved in the type 2 immunity induced pathological process of fibrosis, and complications in CHB also includes the development of fibrosis, cirrhosis, and further progression towards HCC, we examined if the frequencies of CCR4+ CD8+ T cells varies between different stages of fibrosis. Different stages of fibrosis were defined by using FibroScan scores. We monitored higher frequencies of CCR4+ CD8+ T cells in patients with higher fibrosis scores (F3–F4) than those who had lower scores (F0-F2) (Fig. 1F). Similarly, the frequencies of CCR4+ CD4+ T cells were also increased in CHB patients who had higher fibrosis scores (F3-F4), suggesting CCR4 expression increases with disease progression. Further, we checked if CCR4+ CD8+ T cells express any other chemokine receptors including CXCR3, CXCR5, CCR6 and CCR10 and we observed that these cells express all these chemokine receptors, where the former 2 were highly expressed in CHB in comparison to Vacc-HC (Fig. 1G, H). We further compared if there is any difference in the expression of these chemokine receptors between CCR4+ and CCR4- CD8 T cells and we noticed that CCR4+ CD8+ T cells selectively exhibit higher expression of CXCR3, CXCR5 and CCR6 than those of CCR4−CD8+ T counterpart while CCR10 expression did not vary between both the subsets.

It is well established that CHB patients possess exhausted conventional CD8+ T cells owing to the higher expression of inhibitory receptors on these cells; hence, we speculated that CCR4+CD8+ T cells might also express inhibitory receptors. Our results demonstrated that these cells exhibit higher expression of several inhibitory receptors including programmed death-1 (PD-1), cytotoxic T lymphocyte antigen-4 (CTLA4), T-cell immunoreceptor with immunoglobulin and ITIM domains (TIGIT), T cell immunoglobulin and mucin domain-containing protein 3 (TIM 3) except 2B4, which was superior in CCR4−CD8+ T cells. When the profile of these inhibitory receptors was compared between CHB and Vacc-HC, the former group consisted of higher expression of these receptors on both CCR4+ and CCR4−CD8+ T cells, suggesting these cells emulate conventional CD8 T cells in terms of expressing inhibitory receptors (Fig. 1I, J), Next, we determined the activation and proliferation status of CCR4+ CD8+ T cells by analyzing the expression of CD69, an early activation marker, and Ki-67, a marker of cell proliferation. The data revealed that these cells are highly activated and have better proliferation capacity than those of their CCR4−CD8+ T counterpart (Fig. 1K). Overall, these findings demonstrate that CCR4 expression increases with the disease progression and HBs antigen triggers their expression in CD8 T cells. Moreover, CCR4+ CD8+ T cells in CHB are phenotypically distinct from its CCR4−CD8+ T cells counterpart and Vacc-HC.

### CCR4+CD8+ T cells maintain immunosuppressive profile in CHB patients

**Fig. 2:**
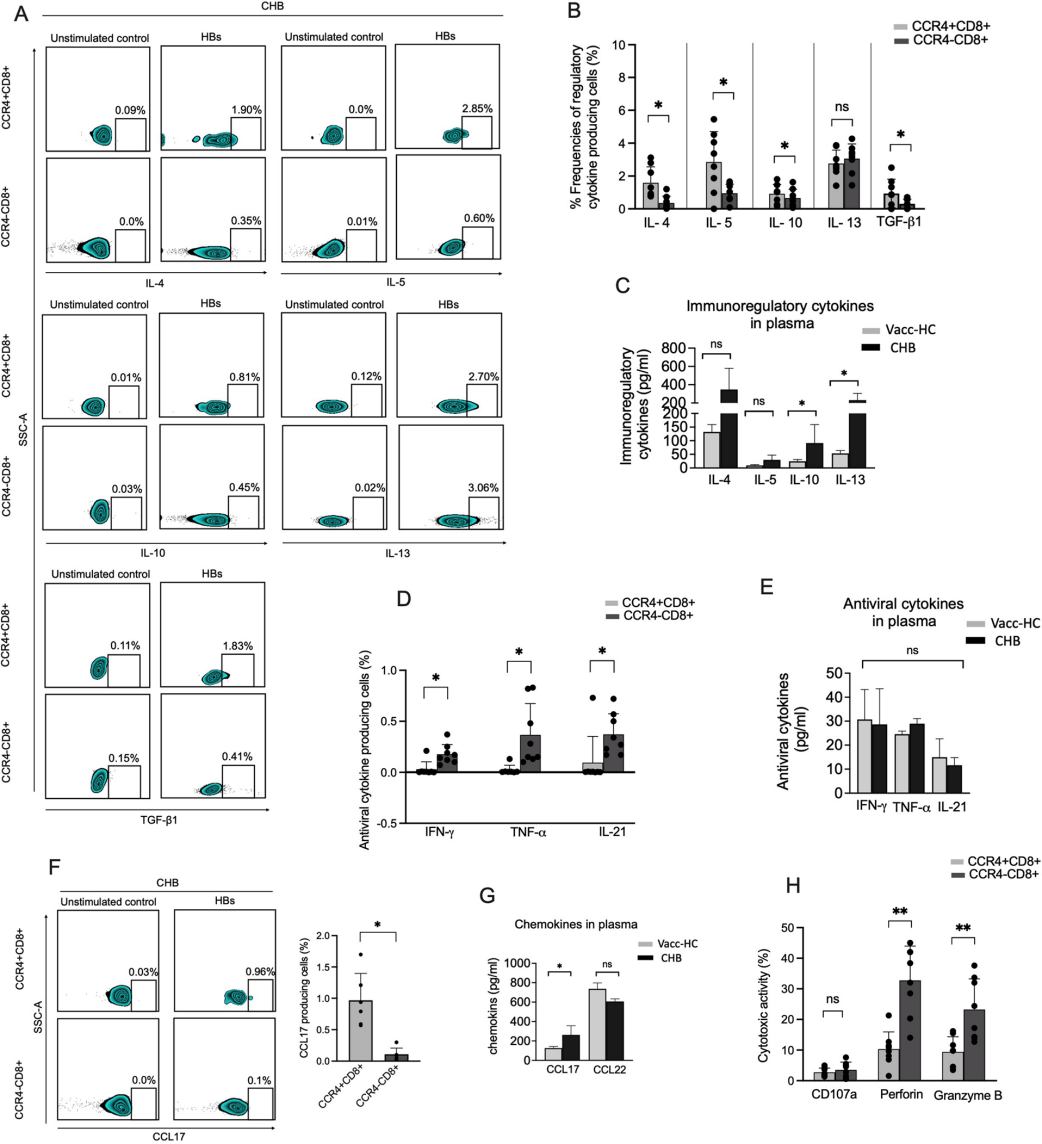
CCR4 + CD8 + T cells retain immunosuppressive profiles in CHB. **A**, **B** Descriptive Zebra plots (**A**) and pooled data in the graphs (**B**) elucidate the percent frequencies of immunoregulatory/immunosuppressive cytokine (IL-4, IL-5, IL-10, IL-13 and TGF-β1) secreting CCR4+CD8+ T cells in CHB patients (n = 8) after 5 days of HBs overlapping peptide stimulation and its comparison with CCR4−CD8+ T cells under in vitro setting. Cells with no HBs overlapping peptide stimulation were taken as unstimulated controls. **C** Plasma levels of immunoregulatory/immunosuppressive cytokines in CHB and Vacc-HC (n = 8 subjects, in each group), measured by multiplex cytokine bead array assay using Luminex technology. **D** Collective data in the scatter plot showcase antiviral cytokine (IFN-γ, TNF-α and IL-21) producing CCR4 + and CCR4- CD8 + T cells in CHB patients after 10 days of PBMC stimulation and expansion with HBs overlapping peptides and IL-2. IL-2 was also added in unstimulated controls (n = 8). **E** Levels of antiviral cytokines in the plasma of CHB and Vacc-HC (n = 8 subjects, in each group). **F** Flow cytometry and scatter plots define chemokine CCL17 producing CCR4+CD8+ T cells in CHB patients (n = 5) and its comparison with its CCR4−CD8+ T cell counterpart after 10 days of PBMC stimulation with HBs peptides and IL-2. **G** Chemokines levels in the plasma of CHB and Vacc-HC. **H** Summary data in scatter plots reveal cytotoxic function (CD107a expression and perforin and granzyme B production) of CCR4+CD8+ T cells in CHB patients after PBMC stimulation with HBs peptides and IL-2 for 10 days. *p < 0.05, **p < 0.01, ***p < 0.001 and ****p < 0.0001

As CCR4+CD8+T cells have not been studied during CHB infection, we thoroughly investigated the functional profile of these cells and compared them to those of their counterpart, CCR4−CD8+ T cells. We examined the production of immunosuppressive/immunoregulatory cytokines comprising IL-4, IL-5, IL-10, IL-13 and TGF-β1 following 5-day stimulation of PBMCs with HBsAg-specific overlapping peptides and we found that CCR4+CD8+ T cells produce higher amounts of these cytokines in response to HBsAg peptide stimulation than those of CCR4−CD8+ T cells in CHB (Fig. 2A, B), suggesting that these cells are immunosuppressive in nature and may associate with immune tolerance in CHB. CCR4+CD8+ T cells in Vacc-HC also displayed higher production of immunosuppressive cytokines than those of their CCR4−CD8+ counterparts; however, it was much lower in comparison to CHB patients (data not shown). Plasma of CHB patients also confirmed elevated levels of these cytokines than Vacc-HC confirming immunosuppressive state in CHB patients (Fig. 2C). Next, we determine whether CCR4+ CD8+ T cells are solely immunosuppressive in nature, or if they are also capable of producing antiviral cytokines like conventional CD8+ T cells. For this experiment, we expanded PBMCs in the presence of HBsAg overlapping peptides and IL-2 for 10 days and checked the production of antiviral cytokines by these cells. The data demonstrated that CCR4+CD8+ T cells primarily do not secrete HBsAg-specific antiviral cytokines including IFN-γ, TNF-α and IL-21 in CHB patients, while their CCR4- counterpart do secrete these cytokines. Out of eight patients, only one showed the production of these cytokines, on the other hand, CCR4−CD8+ T cells produced these cytokines in all the patients (Fig. 2D), signifying that in CHB CCR4+CD8+ T cells constitute immunoregulatory phenotype. However, the plasma levels of these cytokines did not vary significantly between CHB and Vacc-HC (Fig. 2E), which could be due to the fact that plasma cytokines not only constitute the HBsAg-specific cytokines but also represent a global cytokine pool that is not hampered by the HBV. The ligands of CCR4 include chemokine ligands 17 (CCL17) and CCL22 that are majorly produced by the dendritic cells and macrophages [22]. These chemokines are involved in the recruitment of Th2 and T regulatory cells (Tregs), and support type 2 immune response which induce immune suppression in CHB; hence, we decided to check whether CCR4+CD8+T cells contribute to the production of CCL17 in CHB. We noted that these cells are richer in producing CCL17 than those of CCR4−CD8+ T cells (Fig. 2F). In fact, CCL17 level significantly elevated the plasma of CHB patients than Vacc-HC (Fig. 2G), while CCL22 levels were comparable between both the groups.

As conventional CD8 T cells possess cytotoxic function that is essential in the host defense against viral infection, we decipher the cytotoxic capacity in CCR4+CD8+ T cells in CHB patients by analyzing CD107a expression and perforin and granzyme B production after performing 10 days of PBMC culture in the presence of HBs overlapping peptides and IL-2. We observe that these cells have similar levels of CD107a expression as CCR4−CD8+ T cells, while perforin and granzyme B production was markedly lower (Fig. 2H). Collectively, our data reveal that CCR4+CD8+ T cells primarily maintain immunosuppressive profiles in CHB patients by producing higher amounts of immunoregulatory cytokines. Moreover, these cells lack antiviral cytokine response and possess marginal cytotoxic activity.

### Blockade of CCR4 with mogamulizumab antibody boost HBsAg-specific antiviral immune response in T cells

**Fig. 3:**
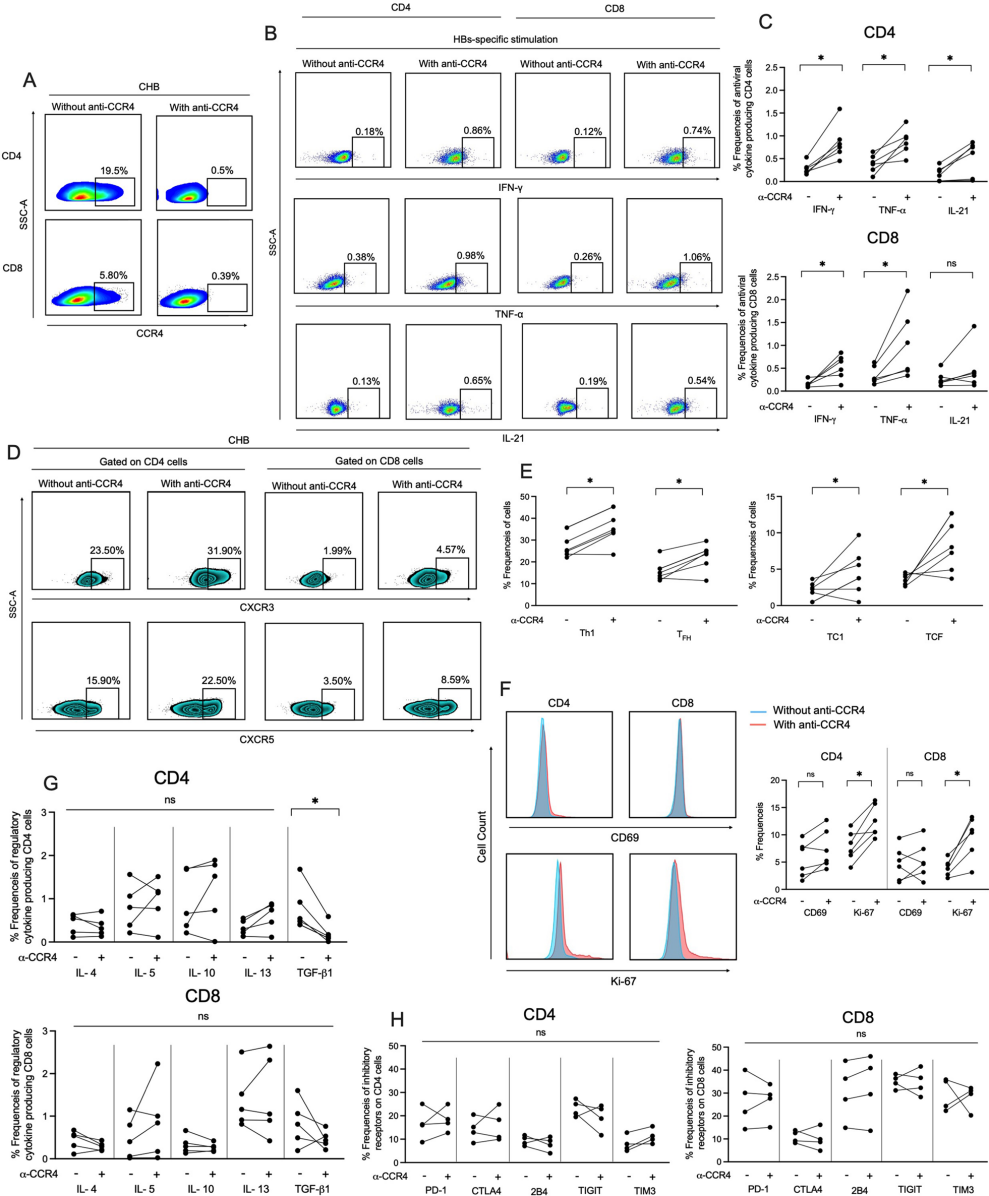
Blockade of CCR4 using Mogamulizumab, an anti-CCR4 antibody, enhances HBsAg-specific antiviral immune response in T cells of CHB patients. **A** Representative flow cytometry figure shows the expression of CCR4 with and without mogamulizumab antibody (20 µg/ml). **B**, **C** Flow cytometry plot and line graphs showcase the production of antiviral cytokines by both CD4 and CD8 T cells in the presence and absence of anti-CCR4 antibody after 10 days of PBMC culture with HBs (n = 6 patients) overlapping peptides along with IL-2. **D**, **E** Analysis of CXCR3 and CXCR5 expression on CD4 and CD8 T cells with and without CCR4 blockade to detect the change in the frequencies of Th1 and T_FH_ cells (in case of CD4) and TC1 and TCF cells (in case of CD8) in CHB patients. **F** Histograms and line graphs showing the expression of CD69 and Ki67 on CD4 and CD8 T cells with and without CCR4 inhibition in CHB patient’s PBMC samples. **G** Line graphs depict the percent frequencies of immunoregulatory/immunosuppressive cytokine producing CD4 and CD8 T cells after 5 days of PBMC culture in the presence of HBs overlapping peptides with and without CCR4 blockade using anti-CCR4 antibody. **H** Expression analysis of inhibitory receptors on CD4 and CD8 T cells after CCR4 blockade. *p < 0.05, **p < 0.01, ***p < 0.001 and ****p < 0.0001

HBV-specific T cell responses are critical in controlling HBV infection; however, these responses are weak in CHB patients, and HBsAg is advocated as one of crucial factors associated with impaired immune responses [23]. Attainment of robust HBsAg-specific immune response associated with viral clearance and attributed to achieve functional cure; therefore, we specifically examined whether anti-CCR4 treatment with mogamulizumab antibody had any impact on the recovery of HBsAg-specific T cell functions in CHB patients. For this, we first identified the optimum concentration of the mogamulizumab antibody to perform CCR4 blockade assay by incubating the PBMCs with different concentrations of anti-CCR4 antibodies (1, 10 and 20 µg) for 5–10 days and then stained the cells with CD3, CD4, CD8 and CCR4 antibodies, and analyzed the expression of CCR4 by flow cytometry (Details provided in supplementary material). We observed that 20 µg/ml mogamulizumab antibody was optimal to block CCR4 receptors (Fig. 3A), and hence used this concentration in the all the CCR4 blockade assays. Next, we assessed HBsAg-specific antiviral cytokine production by both CD4+ and CD8+ T cells following a 10-day expansion of PBMCs with and without HBsAg-specific overlapping peptides and IL-2 in the presence and absence of anti-CCR4 antibody. We observed a marked increase in IFN-γ and TNF-α production by CD4+ and CD8+ T cells after CCR4 blockade while IL-21 production was enhanced only in CD4 T cells. Representative flow cytometry plots for CD4+ as well as CD8+ T cell responses are depicted in figure (Fig. 3B) and cumulative data presented in the line plots (Fig. 3C). IFN-γ and IL-21 producing cells were more frequently detectable in CD4 cell compartment than those of CD8+ T cells, whereas TNF-α producing cells were higher in CD8 T cell compartment. Unstimulated controls showed minimal increase in antiviral cytokine secretion after CCR4 blockade (data not shown). Additionally, we checked if CCR4 blockade could enhance antiviral cytokine secretion against HBcAg and we found augmentation of HBcAg-specific antiviral cytokine secretion in both CD4 and CD8 T cells (Additional file 1: Fig. S2). Since IFN-γ and TNF-α are primarily produced by T helper type 1 (Th1) and cytotoxic T cell type 1 (TC1) cells whereas IL-21 is secreted by T follicular helper (T_FH_) cells and cytotoxic T follicular (T_CF_), we investigated if CCR4 blockade shifts CD4+ and CD8+ T cells towards these phenotypes. Interestingly, we observed that CCR4 blockade polarized CD4+ and CD8+ T cells towards Th1 and T_FH_ cells in case of CD4+ cells, and TC1 and T_CF_ cells in CD8+ T cell population (Fig. 3D, E), which could be possibly through inducing higher proliferation in these cells as we monitored higher expression of Ki-67 in both CD4 and CD8 T cells after CCR4 blockade (Fig. 3F). In addition, we investigated if anti-CCR4 treatment could prompt early activation in CD4+ and CD8+ T cells; however, we did not witness any such changes after CCR4 blockade (Fig. 3F). Overall, these findings suggest that anti-CCR4 treatment reverses the functional impairment of HBsAg-specific CD4+ and CD8+ T cells and boost antiviral immune response in these patients that are needed for viral elimination.

Since the degree of T cell impairment is also governed by the levels of immunosuppressive cytokines, we questioned whether anti-CCR4 treatment amplifies the production of immunosuppressive cytokines against HBsAg, which will restrict effector T cell responses. We tested the production of immunosuppressive cytokines including IL-4, IL-5, and IL-10, IL-13 and TGF-β1 by CD4+ and CD8+ T cells with and without anti-CCR4 treatment and found that blockade of CCR4 did not encourage the production of any of the immunosuppressive cytokines in both CD4+ and CD8+ T cells (Fig. 3G). In fact, inhibition of CCR4 declines the production of HBsAg-specific TGF-β1 in CD4 cells; therefore, may limit the immune tolerance and eliminates the risk of anti-CCR4 treatment induced antiviral immune suppression.

Given that immune checkpoint blockade of inhibitory receptors recovers HBV-specific T cell response, we examined if anti-CCR4 treatment could suppress the expression of these inhibitory receptors on CD4+ and CD8+ T cells. We analyzed the expression of multiple inhibitory receptors including PD-1, CTLA4, 2B4, TIGIT and TIM3 on both CD4+ and CD8+ T cells with and without CCR4 blockade; however, we did not observe any reduction in the expression of these inhibitory receptors on both CD4+ and CD8+ T cells after CCR4 blockade (Fig. 3H), indicating CCR4 might not be associated with the expression of inhibitory receptors and hence did not alter the expression of these receptors upon its blockade.

### Anti-CCR4 treatment with mogamulizumab antibody improves HBsAg-specific cytotoxic function in T cells

**Fig. 4:**
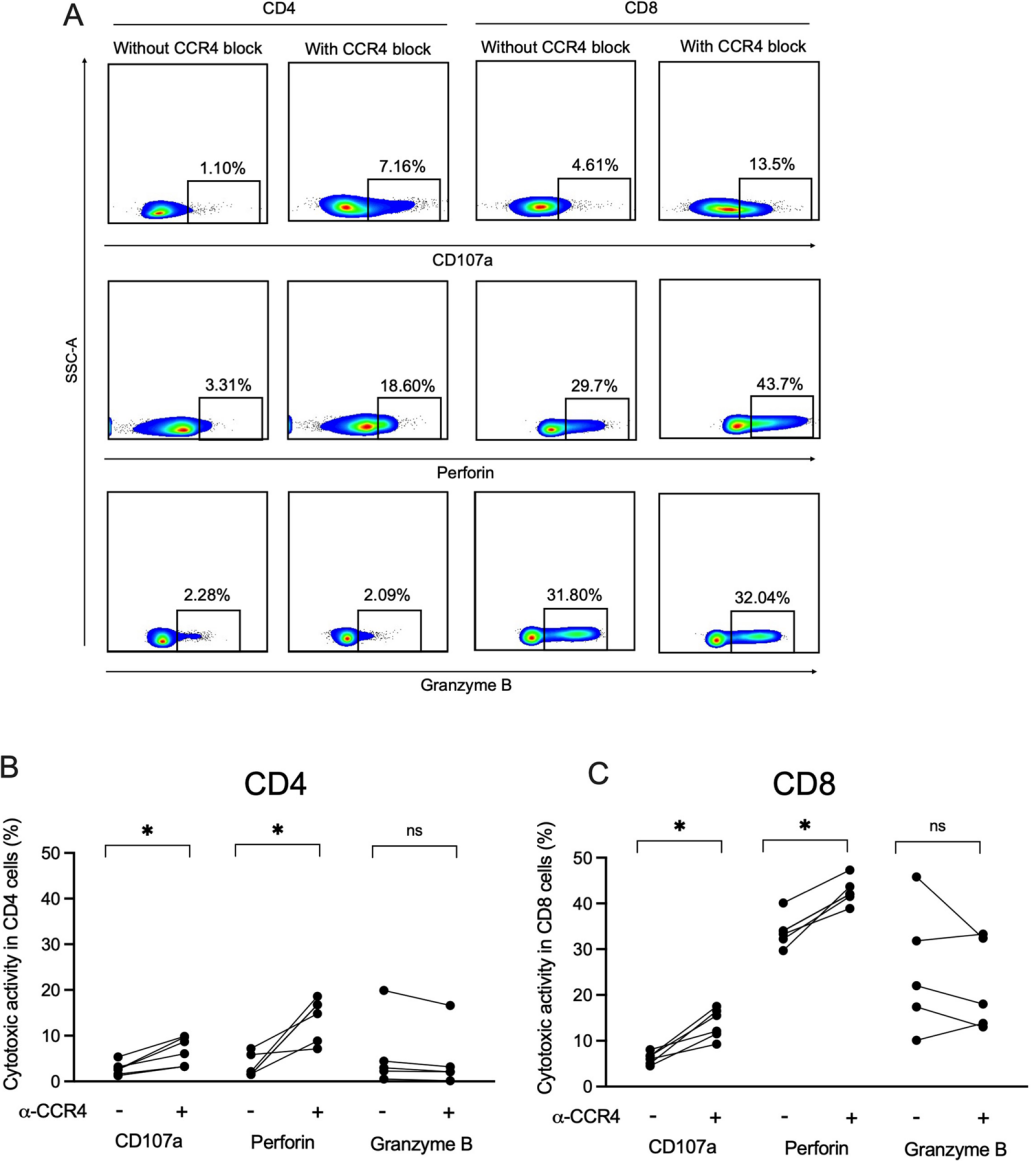
Treatment with anti-CCR4 antibodies enhances cytotoxic function in T cells of CHB. **A** Example flow cytometry plots demonstrating the effect of CCR4 blockade on the recovery of cytotoxic potential in CD4 and CD8 T cells after 10 days of PBMC stimulation and expansion with HBs overlapping peptides and IL-2 in the presence and absence of anti-CCR4 antibody. **B**, **C** Line graphs describe the cytotoxic function (CD107a expression, perforin and granzyme B) in CD4 and CD8 T cells of CHB patients with and without CCR4 inhibition. *p < 0.05

Cytotoxic T cells can kill the virus-infected cells by expressing degranulation marker CD107a and releasing cytotoxic proteins including perforin and granzyme B. These proteins induce apoptosis pathway for effective killing of virus-infected cells by effector CD8+ T cells and these functions shown to be reduced in CHB patients. Therefore, we determined if anti-CCR4 treatment could boost cytotoxic function of T cells. Classically, CD8 T cells are known to possess cytotoxic function; however, in the recent times it has been identified that a subset of CD4+ T cells also have cytotoxic properties as shown by their ability to secrete perforin and granzyme B to kill the target cells in an MHC class II-restricted manner [24]. Consequently, we examined the cytotoxic functions in both CD4+ and CD8+ T cells. Given that these proteins are also expressed in spontaneous condition, we stimulated the cells with HBsAg-specific overlapping peptides and then checked the expression of these molecules. We found that CD4+ T cells in CHB patients possess cytotoxic functions as shown by CD017a expression and perforin and granzyme B production; although, they were much lower than those of CD8+ T cells and marginally increased in response to HBsAg (Fig. 4A). Notably, CCR4 blockade significantly augmented the frequency of CD107a+ cells and perforin production in both CD4+ and CD8+ T cells; though, granzyme B production did not improve (Fig. 4A–C), demonstrating that blockade of CCR4 is beneficial in improving overall T cells functions including antiviral cytokine response and cytotoxic potential that might help in HBV clearance.

### Blockade of CCR4 inhibits the development and effector function of T regulatory cells

**Fig. 5:**
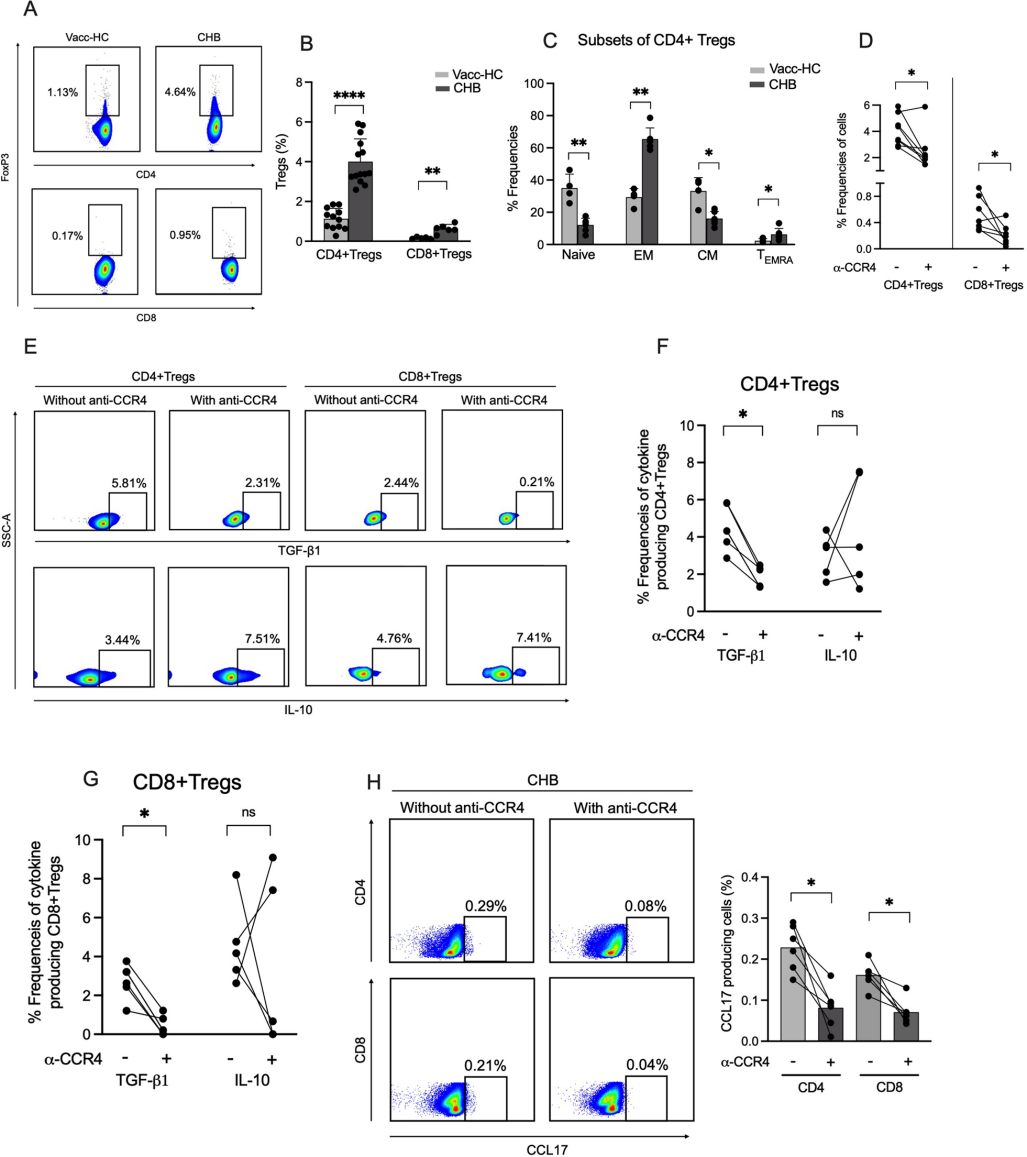
Neutralization of CCR4 prevents the development and effector function of T regulatory cells in CHB. **A**, **B** Ex vivo analysis of the percentage of CD4 T regulatory cells (CD4Tregs) and CD8Tregs in the PBMCs of Vacc-HC and CHB patients. **C** Frequencies of different subsets of CD4Tregs (Naïve: CCR7+CD45RO−, EM: CCR7−CD45RO+, CM: CCR7+CD45RO+, T_EMRA_: CCR7-CD45RO−) in CHB patients and its comparison with Vacc-HC under ex vivo conditions. EM, effector memory; CM, central memory; T_EMRA_, terminally differentiated effector memory. **D** The percentage of Tregs after 5 days of PBMC culture in the presence of HBs overlapping peptides with and without CCR4 neutralization using anti-CCR4 antibody. **E**–**G** Representative flow pictures (**E**) and line graphs (**F**, **G**) illustrate the frequencies of immunosuppressive cytokine (IL-10 and TGF-β1) producing Tregs with and without CCR4 inhibition after 5 days of culture in the presence of HBs peptides. **H** CCL17 production in response to HBs peptide in the presence and absence of CCR4 blockade. *p < 0.05, **p < 0.01, ***p < 0.001 and ****p < 0.0001

Regulatory T cells are a specialized subpopulation of T cells that suppresses the immune response by inhibiting T cell proliferation and cytokine secretion to maintain homeostasis and self-tolerance. However, aggravated response by Tregs can dampen T cell immunity leading to viral persistence. In CHB, Tregs are linked to an impaired immune response. Our data demonstrated increased frequencies of CD4+FoxP3+Tregs in CHB patients (Fig. 5A, B), which is in agreement to the previous studies [25]. These CD4 + FoxP3 + Tregs consisted lower percentage of central memory (CM) as well as naïve cell population and were mostly enriched in effector memory (EM) and terminally differentiated effector memory (T_EMRA_) cells (Fig. 5C), which is also reflected by higher amount of IL-10 and TGF-β1 production by these cells in CHB patients (Fig. 5E), confirming the expansion of functional effector Tregs in these patients. Recently, the existence of CD8+ Tregs have been discovered in different experimental systems. Analogous to CD4+ Tregs, these cells also carry certain regulatory properties and participate in immune regulation; therefore, we investigated whether CHB patients also carry CD8+ Tregs. Our data revealed that CHB patients do possess CD8+FoxP3+Tregs and indeed retain higher frequencies than those of Vacc-HC (Fig. 5A, B). Since, Tregs play a critical role in immune suppression in CHB patients and are associated with viral persistence in these patients; we investigated whether inhibition of CCR4 can control the immune suppressive function of Tregs. Notably, we observed a significant decline in the frequencies of CD4+ and CD8+ Tregs after the CCR4 blockade (Fig. 5D). Importantly, HBsAg-specific TGF-β1 production was also diminished by these cells, while IL-10 production remained stable (Fig. 5E–G). Reduction in Tregs and TGF-β1 production after anti-CCR4 treatment advocated that CCR4 is required for the development and effector function of Tregs and its blockade potentially subsidize HBsAg-specific immunoregulatory functions of Tregs that might benefit CHB patients in term of controlling immunosuppressive environment in these patients. CCL17, a ligand of CCR4 enables Tregs recruitment to the site of infection where they induce immune suppression by TGF-β1 production; hence we investigated if CCR4 blockade could control HBsAg-specific CCL17 secretion by T cells and we found a significant reduction in CCL17 production post CCR4 blockade, which might further benefit in restricting Tregs effector function in CHB patients (Fig. 5H). Moreover, CCL17 production against HBcAg was also diminished upon CCR4 blockade (Additional file 1: Fig. S3).

## Discussion

In the present study, we report that blockade of CCR4 with mogamulizumab antibody effectively recovers antiviral immune response and limits immune suppression mediated by Tregs that might be helpful in HBV clearance. One of the main hurdles to HBV clearance in CHB patients is a dysfunctional adaptive immunity, characterized by intense exhaustion of HBV-specific T cells. Generally, HBV-specific CD8+ cytotoxic T cells are considered to be central player in the eradication of HBV infection, which is confirmed by the depletion of CD8+ T cells at the peak of viremia in experimentally infected chimpanzees that delayed the viral clearance until the recovery of CD8+ T cell [26]. In chronic HBV infected patients, HBV-specific CD8 T cell responses are quantitatively and qualitatively weak and there is unanimity that effector CD8 T cells have various levels of exhaustion such as (1) reduced proliferation capacity, (2) decreased antiviral cytokine production, (3) poor cytotoxic function and (4) persistently higher expression of inhibitory receptors. In addition, HBV-specific CD4+ T cell functions are also impaired, hindering the activation and maintenance of the HBV-specific CD8+ T cells, and generation of HBV-specific humoral responses [27]. Therefore, we identified whether treatment with anti-CCR4 antibody could help in the reversal of various levels of immune exhaustion in CHB patients. Generally, CCR4 is expressed on Th2 and Tregs subsets of CD4+ T cells that are primarily associated with immune regulation; however, hyper-activation of these cells leads to the development of immune tolerance in CHB. Our results demonstrated that in CHB the frequency of CCR4 expressing cells is higher in both CD4+ as well as CD8+ T cell compartment, which could be due to their higher proliferation as we found more Ki-67 expression on these cells. As CCR4+CD4+ T cells have already been characterized in different disease settings and there is inadequate data available on the role of CCR4 on CD8 T cells during CHB, we first focused on the characterization of CCR4+CD8+ T cells and identified that these cells basically possess immunoregulatory function during chronic HBV infection. Unlike, conventional CD8+ T cells which produce higher amounts of IFN-γ and TNF-α, CCR4+CD8+ T cells typically produce immunosuppressive cytokines including IL-4, IL5, IL10 and IL-13 and TGF-β1 and lack antiviral cytokine response, although they retain partial cytotoxic function than those of CCR4−CD8+ T cells, confirming regulatory role of these cells during CHB. Importantly, CCR4+CD8+ T cells progressively increase in patients with higher HBsAg levels and fibrosis scores proposing their role in disease progression. In fact, in vitro data revealed that HBsAg could directly trigger CCR4 expression in CD8 T cells. CCR4 expressing Th2 cells have already been reported to be involved in fibrosis via IL-4 and IL-13 secretion [28, 29]. Our finding of IL-4 and IL-13 secretion along with other pro-fibrotic cytokine TGF-β1 production by CCR4+CD8T not only suggest the role of these cells in fibrosis b9ut endorse that these cells might have clinical significance. In addition, CCR4+CD8+ T cells present several inhibitory receptors whose expression is markedly higher in comparison to CCR4−CD8+ T cells, linking their contribution to the exhaustion of conventional CD8 T cells.

Under in vitro setting, blockade of CCR4 revealed promising data by showing its potential to reverse various levels of immune dysfunction in both CD4 and CD8 T cells that we have discussed in the following points.

**Fig. 6:**
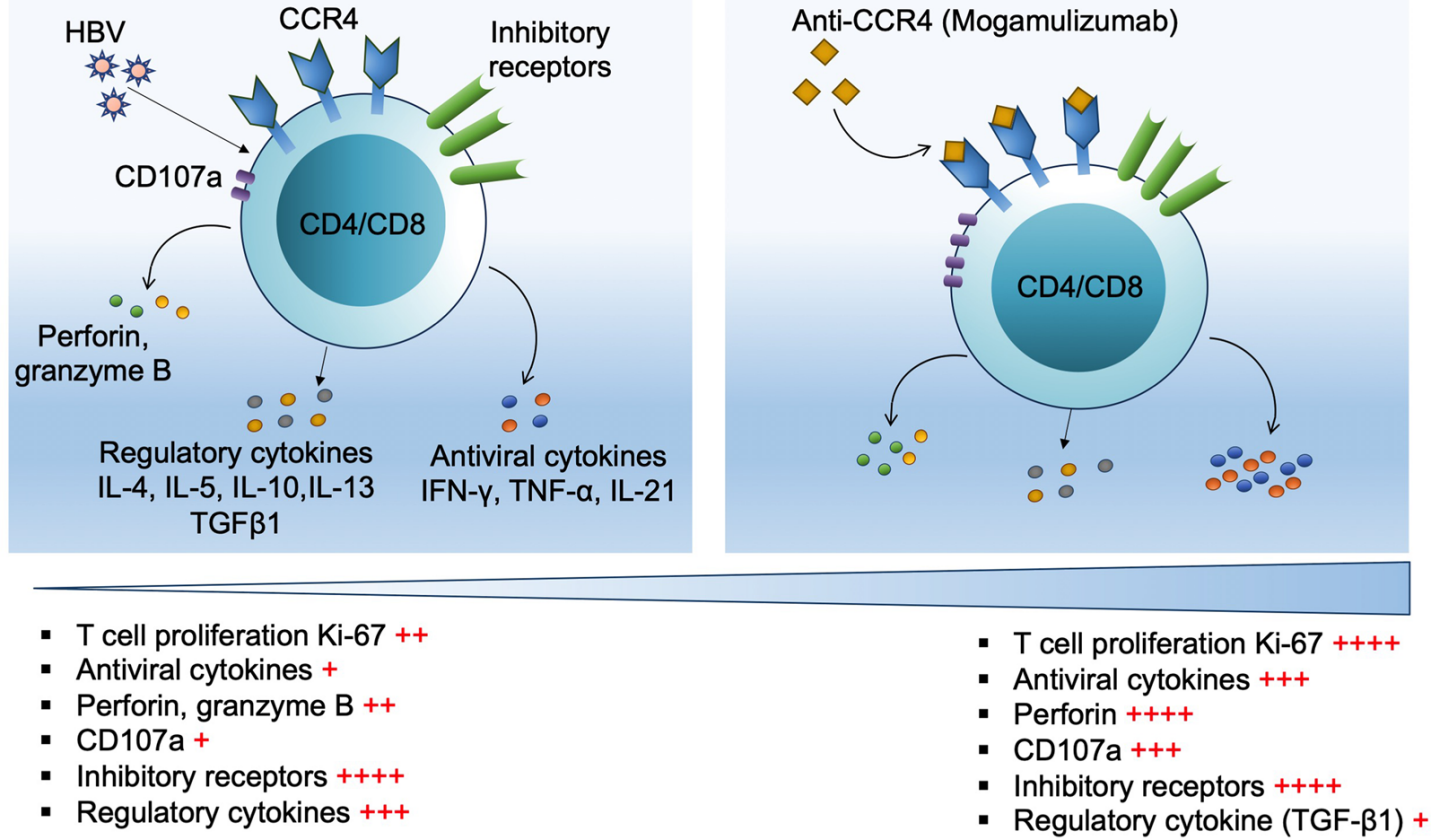
Hepatitis B virus infection induces **i**mmunosuppression and functional exhaustion of effector T cells driving persistent chronic hepatitis B virus (HBV) infection. CCR4 expressing T cells contributes to the immunosuppression by limiting the HBV-specific antiviral response in T cells and supporting Tregs functions. Blockade of CCR4 reverses immune exhaustion in T cells and limits immunosuppressive activity of Tregs

1. T cell proliferation: T cell proliferation is a critical step for the clonal expansion of antigen-specific T cells and their further transition into effector cells, which is a fundamental requirement to mount an effective T cell response [30]. In CHB, T cell proliferation capacity is impaired, mostly against HBsAg, which is even worse in CD8 T cells than those of CD4 subset [31]. Therefore, the first step to achieve functional restoration of antigen-specific T cells is to recover its proliferation capacity and we found that anti-CCR4 treatment improves proliferation capacity of both CD4 and CD8 T cell subsets (Fig. 3F).
2. Antiviral cytokine secretion: After transitioning into effector cells, T cells perform various functions such as cytokine secretion; however, antiviral cytokine secretion is severely impaired in CHB patients especially in response to HBsAg, contributing to persistent viral infection [21]. Considering the fact that antiviral cytokine secretion is one of the most crucial steps for the attainment of successful viral elimination, strategies that help in the improvement of antiviral cytokines response are strongly needed. Blockade of CCR4 remarkably fulfill that criterion by augmenting antiviral cytokines IFN-γ, TNF-α and IL-21 secretion in CD4 and CD8 T cells through diverging these cells towards Th1 and T_FH_ in case of CD4 while TC1 and T_CF_ cells in case of CD8 T cells (Fig. 3B–E). Importantly, enhancement of IL-21 secretion will support the development of HBV-specific humoral responses to generate neutralizing antibodies against HBV. Moreover, blockade of CCR4 did not induce the production of immunosuppressive cytokines that eliminated the possibility of anti-CCR4 treatment induced immune suppression in these patients.
3. Cytotoxic function: Another crucial function performed by effector T cells is to kill virus infected cells that is carried out by the lysosomal-associated membrane protein-1 (Lamp-1), also known as CD107a, and cytotoxic granules including perforin and granzyme B, which mediate activation of apoptotic pathways [32]. These functions are compromised in CHB infection, and its retrieval is another milestone to achieve successful elimination of virus-infected cells. Our data have shown that CCR4 inhibition competently restores the cytotoxic potential of T cells including CD107a expression as well as perforin secretion that will allow effective killing of the virus infected cells and thus will help in reducing the viral load. Overall results have been summarized in Fig. 6.

Additional feature of exhausted T cells is to express multiple inhibitory receptors that derive defective T functions [33]. Studies are focusing on immune checkpoint inhibitors to rescue T cell functions; thus, we tested if treatment with anti-CCR4 antibody could decline the expression of these inhibitory receptors; however, this could not be achieved with CCR4 blockade. The goal of targeting the inhibitory receptors is to achieve functional restoration of T cells that anyway is accomplished by CCR4 blockade; therefore, impose no concerns in this regard. Collectively, our data reveal that CCR4 blockade is effective in the overall recovery of various levels of immune dysfunction in HBsAg-specific T cells including both CD4 and CD8 T cells that might help in HBV clearance.

Another mechanism contributing to the suppression of HBV-specific immunity is the expansion of the immune tolerogenic/immunosuppressive environment that is majorly driven by Tregs. In general, Tregs maintain immune equilibrium by suppressing the antigen-specific or antigen non-specific T cell responses using various mechanisms including IL-10 and TGF-β1 production; though, higher expansion of Tregs induce immunosuppression resulting in viral persistence [25]. Therefore, depletion of Tregs might be a good approach for the recovery of effector T cell functions. In fact, studies have shown that downregulation in the proportion of circulating Tregs is helpful in restoring the antiviral immune response [34]. Our data reported higher frequencies of Tregs in CHB and these cells were mainly enriched in effector and terminally differentiated effector memory cells and produced higher amounts IL-10 and TGF-β1. These cytokines not only cause immunosuppression but are strongly associated with further disease progression into fibrosis, cirrhosis, and hepatocellular carcinoma in CHB. Strikingly, CCR4 blockade declined the frequencies and function of Tregs. TGF-β1, a critical molecule that participates in fibrosis progression and in HCC development, dropped significantly post CCR4 blockade, suggesting CCR4 as a critical target to control Tregs mediated immune suppression and progression of liver disease.

Upregulated CCR4 expression has been observed in several types of cancers that associate with tumor growth, invasion, migration and even metastasis serving them as a critical target in various cancers including HCC [17, 28, 35]. In addition to CCR4, its ligands CCL17 and CCL22 found to be elevated in liver cancer, which facilitate Tregs cell infiltration to the tumors leading to the suppression of anti-cancer immunity by TGF-β1 production, and further establishment of a favorable microenvironment for tumor growth [36, 37]. Our data reported a reduction in CCL17 production after CCR4 blockade which in turn will impede Tregs function including TGF-β1 production and thereby, restraining immune immunosuppression in these patients. Given that CCR4 is a critical player in various cancers such as HCC, anti-CCR4 antibodies including mogamulizumab are being used to enhance anti-cancer immunity [38]. Our data revealed an important finding that CCR4 expression is not directly upsurge during end-stage liver disease such as HCC, but it tends to increase in the early phase of the disease such as CHB and augment with disease progression; therefore, blockade of the CCR4 in an earlier stage could be more beneficial to control disease progression towards end-stage disease, at least in case of CHB infection. CHB patients most often require long-term treatment with nucleos(t)ide analogues (NAs) which put them at risk of developing drug resistance [39]. In addition, discontinuation of the NAs may lead to HBV reactivation [40]; hence, therapeutic strategies targeting to achieve functional cure in short period of time may avoid complications associated with the long-term use of NAs and combination therapy of NAs with anti-CCR4 could be considered as an option to treat these patients.

In conclusion, our study provides a novel and significant information about the role CCR4 during CHB infection and demonstrate that CCR4 is a major player in HBV-specific T cell immune dysfunction in CHB patients and its blockade recovers various levels of immune dysfunction such as cell proliferation, antiviral cytokine secretion and cytotoxic T cell functions. Besides, CCR4 inhibition limits the phenotype and function of Tregs that will further help in improving antigen-specific T cell functions required for viral elimination, and therefore CCR4 could serve as a potential therapeutic target for the treatment of CHB patients.

## Supplementary Information

**Additional file 1:** **Material and Methods S1.** **Table S1.** **Figure S1:** Hepatitis B core antigen (HBc) induces CCR4 expression in CHB patients. Line graph represents the expression of CCR4 on CD8 T cells in CHB patients after PBMC stimulation with HBc peptides for 5 days. PBMCs without any HBc stimulation were taken as controls. **Figure S2.** Blockade of CCR4 with Mogamulizumab, an anti-CCR4, boosts HBcAg-specific immune response in T cells of CHB patients. (A, B) Flow cytometry plot and line graphs designate the production of antiviral cytokines by CD4 and CD8 T cells in the presence and absence of anti-CCR4 antibody after 10 days of PBMC stimulation with HBc overlapping peptides along with IL-2. **Figure S3.** Blockade of CCR4 with Mogamulizumab inhibits CCL17 production by T cells. Line graph indicates the production of CCL17 upon HBc peptide stimulation for 10 days along with IL-2, in the presence and absence of anti-CCR4 antibody.

## Data Availability

All the data generated and utilized in this study are included in this published article.

## Abbreviations

CHB: Chronic hepatitis B
CCR/CXCR: Chemokine receptor
CCL: Chemokine ligand
CTLA4: Cytotoxic T lymphocyte antigen-4
CM: Central memory
EM: Effector memory
FBS: Fetal bovine serum
FOXP3: Forkhead box P3
HBV: Hepatitis B virus
HBsAg: Hepatitis B surface antigen
HBeAg: Hepatitis B e antigen
HCC: Hepatocellular carcinoma
IL: Interleukin
IFN-γ: Interferon gamma
NAs: Nucleos(t)ide analogues
PBMCs: Peripheral blood mononuclear cells
PD-1: Programmed death 1
RPMI 1640: Rosewell Park Memorial Institute
ROS: Reactive oxygen species
TLR8: Toll-like receptor 8
TNF-α: Tumor necrosis factor alfa
TGF-β1: Transforming growth factor beta-1
Tregs: T regulatory cells
TFH: T follicular helper
TCF: T cytotoxic follicular
Th: T helper
TC: T cytotoxic
TIGIT: T-cell immunoreceptor with immunoglobulin and ITIM domains
TIM-3: T-cell immunoglobulin and mucin-domain containing-3
T_EMRA_: Terminally differentiated effector memory
