# Supplementary material for: Blockade of CCR4 breaks immune tolerance in chronic hepatitis B patients by modulating regulatory pathways
Source: J Transl Med. 2023 Apr 21;21:271. doi: 10.1186/s12967-023-04104-8 (PMC10120209; doi:10.1186/s12967-023-04104-8)
Supplement: Supplementary file 1 — Additional file 1: Material and Methods S1. Table S1. Figure S1: Hepatitis B core antigen (HBc) induces CCR4 expression in CHB patients. Line graph represents the expression of CCR4 on CD8 T cells in CHB patients after PBMC stimulation with HBc peptides for 5 days. PBMCs without any HBc stimulation were taken as controls. Figure S2. Blockade of CCR4 with Mogamulizumab, an anti-CCR4, boosts HBcAg-specific immune response in T cells of CHB patients. (A, B) Flow cytometry plot and line graphs designate the production of antiviral cytokines by CD4 and CD8 T cells in the presence and absence of anti-CCR4 antibody after 10 days of PBMC stimulation with HBc overlapping peptides along with IL-2. Figure S3. Blockade of CCR4 with Mogamulizumab inhibits CCL17 production by T cells. Line graph indicates the production of CCL17 upon HBc peptide stimulation for 10 days along with IL-2, in the presence and absence of anti-CCR4 antibody. [file 12967_2023_4104_MOESM1_ESM.docx]

**Blockade of CCR4 Breaks Immune Tolerance in Chronic Hepatitis B Patients by Modulating Regulatory Pathways**

**Authors:** Arshi Khanam*, Alip Ghosh, Joel V Chua, Shyam Kottilil

**Address**

Division of Clinical Care and Research, Institute of Human Virology, University of Maryland School of Medicine, Baltimore, MD, USA

Table of contents

Material and Methods S1 ……………………....... 2-6

Table S1……………………....................... 7

Fig. S1……………………....................... 8

Fig. S2…………………………………….. 9

Fig. S3 …………………………………….. 10

***Material and Methods:***

***Flow Cytometry Analysis***

PBMCs collected from Vacc-HC and CHB patients were thawed and rested overnight in complete RPMI 1640 medium supplemented with 10% FBS (GIBCO), 1% penicillin and streptomycin (Sigma-Aldrich), and 1% glutamine (Sigma-Aldrich) at 37^0^ C in a CO2 incubator. Next morning, cell count and viability was assessed by Trypan blue staining. ≥ 90% cells were viable. For surface staining, cells were first stained with Fixable Live/Dead Zombie Aqua stain (1:1000 dilution) for 30 minutes at room temperature. After a PBS wash, cells were incubated with a panel of 16 anti-human monoclonal antibodies against CD3, CD4, CD8, CCR4, CCR6, CCR7, CCR10, CXCR3, CXCR5, CD45RO, programmed death-1 (PD-1), cytotoxic T lymphocyte antigen 4 (CTLA4), T-cell immunoreceptor with immunoglobulin and ITIM domains (TIGIT), T-cell immunoglobulin and mucin-domain containing-3 (TIM-3), 2B4, and CD69 for 20 minutes. Further, intra-nuclear staining with anti-human Ki-67 and FOXP3 antibody was performed after fixation and permeabilization of cells with eBioscience FOXP3/transcription factor staining buffer set (Cat no. 00-5523-00), according to the manufacturer’s instructions. Cells were washed with 1X PBS, fixed in 0.5% paraformaldehyde, and acquired on Cytek Aurora flow cytometer (Cytek Biosciences). Data was analyzed using FlowJo version 10 software (v10; FlowJo, LLC, Ashland, OR). To determine the optimal antibody concentration for staining, all the antibodies were titrated before use. Details of all the antibodies used in this study is provided in the supplementary Table 1.

***Analysis of CCR4 Expression***

To analyze if HBV triggers the expression of CCR4 on CD8 T cells, overnight rested PBMCs from CHB patients were cultured in complete RPMI 1640 medium in 48 well flat bottom plate (1X10^6^ cells) in the presence of HBsAg-specific (PM-HBV-LEPULTRA, a pool of 216 peptides derived from a peptide scan (15mers with 11aa overlap) through large envelop protein of HBV and HBcAg-specific overlapping peptides (PM-HBV-CPULTRA, a pool of 155 peptides derived from a peptide scan (15mers with 11 aa overlap) through Capsid protein of HBV) at a concentration of 1µg/ml or in a medium alone as a control and incubated at 37^0^C in a 5% CO2 incubator for 5 days. On 4^th^ day, cells were re-stimulated with HBs and HBcAg-specific peptides. Next morning, cells were washed and stained with live/dead Zombie Aqua stain for as per above mentioned antibody dilution, followed by surface staining with anti-human CD3, CD8 and CCR4 antibodies for 20 minutes at room temperature. Cells were then washed with 1X PBS and fixed in 0.5% paraformaldehyde for flow cytometry analysis.

***Detection of Immunoregulatory/Immunosuppressive Cytokines***

To examine the production of immunoregulatory/immunosuppressive cytokines, PBMCs from CHB patients were stimulated with HBsAg-specific overlapping peptides (1µg/ml) in 48 well flat bottom plate (1X10^6^ cells) for 5 days. Cells without any HBs stimulation were taken as controls. On day 4, cells were re-stimulated with HBsAg-specific peptides and golgi plug having Brefeldin A (1 µg/ml, BD Biosciences, San Diego, CA) was added after 2 hours of incubation. Next day, cells were washed, and live/dead staining was performed with Zombie Aqua stain followed by surface staining with a cocktail of anti-human CD3, CD4, CD8, CCR4 antibodies and further intracellular staining was executed with IL-4, IL-5, IL-10, IL-13 and transforming growth factor-β1 (TGF-β1)-anti-human antibodies after fixation and permeabilization of cells with cytofix/cytoperm (BD Biosciences). Samples were analyzed by flow cytometry.

***Analysis of Antiviral Cytokines and Degranulation Activity***

For the detection of antiviral cytokines, overnight rested PBMCs (1X10^6^ cells/ml) from CHB patients were cultured in 24 well flat bottom plate in complete RPMI 1640 medium with or without HBsAg-specific overlapping peptides at a final concentration of 1µg/ml and incubated at 37^0^C in a 5% CO2 incubator for 10 days. Recombinant IL-2 (20 IU/ml) from Tecin, Biological Resources Branch, NIH, was added at days 1, 4 and 7. Cells that were cultured without HBsAg-specific peptides were taken as controls; however, IL-2 was still added on the given days to maintain the cell survival in controls. On 9^th^ day, cells were re-stimulated with HBsAg-specific peptides as per the above-mentioned concentration and anti-human CD107a antibody was added (5µl/ml). Later, golgi plug (1 µg/ml) was added after initial 2 hours of incubation. The following day, live/dead staining with Zombie Aqua stain was conducted and then surface staining was performed using anti-human CD3, CD4, CD8 and CCR4 antibodies. Plates were then washed with 1X PBS and centrifuged at 1300 rpm for 5 minutes and intracellular staining with IFN-γ, TNF-α, IL-21, CCL17, perforin and granzyme B was executed after fixation and permeabilization of cells with cytofix/cytoperm. Cell acquisition and data analysis was done as described above.

***Anti-CCR4 Antibody***

Monoclonal anti-CCR4 antibody mogamulizumab (clone: KW-0761) was purchased from abcam (Cat no. ab275980). This chimeric rabbit antibody was prepared by using variable domain sequences of the original human IgG1 format and produced recombinantly to maintain high batch to batch consistency. To find out the optimum concentration of the mogamulizumab for CCR4 blockade, different antibody concentrations were tested. The optimal concentration was investigated by incubating the PBMCs with different concentrations of anti-CCR4 antibodies including 1, 10 and 20 µg for 5-10 days. To maintain the cell survival in 10 days culture, 20 IU/ml of IL-2 was added. Following the completion of the incubation time, cells were stained with CD3, CD4, CD8 and CCR4 antibodies and the expression of CCR4 was analyzed on T cells by flow cytometry. We observed that 20 µg/ml mogamulizumab was optimal to block CCR4 receptors, and hence used this concentration in the final experiments.

***CCR4 Blockade Assay***

To examine the role of CCR4 blockade on diverse T cell functions, PBMCs were cultured in 2 different settings as follows (1) 1X10^6^ PBMCs from CHB patients were cultured in 48 well plate in the presence of HBs and HBcAg-specific peptides (1µg/ml) for 10 days with and without anti-CCR4 antibody mogamulizumab (20µg/ml). Addition of IL-2 (20IU/ml) was carried out on day 1, 4 and 7. HBs and HBcAg-specific re-stimulation of the cells was performed on day 9 and CD107Aa was added (5µl/ml). Following 2 hours of incubation, golgi plug was added at a concentration described earlier. Next day, after live/dead staining, surface and intracellular/intranuclear staining was conducted as per the protocol defined in the earlier experiments using CD3, CD4, CD8, CCR7, CXCR3, CXCR5, PD1, CTLA4, TIGIT, TIM3, 2B4, CD69, perforin, granzyme B, IFN-γ, TNF-α and IL-21, CCL17 and Ki67 anti-human antibodies in different panels. (2) In the next set of experiments, cells were incubated in 48 well plates with HBs-specific peptides (1µg/ml) in the presence and absence of anti-CCR4 antibody mogamulizumab (20µg/ml) for 5 days. Re-stimulation was performed on day 4 using HBs-specific peptides. Addition of golgi plug was done after 2 hours of incubation and staining was carried out according to the protocol explained in earlier experiments using CD3, CD4, CD8 and CCR4 antibodies, followed by the intracellular staining with IL-4, IL-5, IL-10, IL-13 and TGF-β1 antibodies after fixation and permeabilization of cells with cytofix/cytoperm. Acquisition of the samples was performed on Cytek Aurora followed by analysis on FlowJo version 10 software.

***Treatment of Regulatory T Cells with Anti-CCR4 antibody***

To evaluate the role of CCR4 blockade on regulatory T cells (Tregs), overnight rested PBMCs were stimulated with HBsAg overlapping peptides (1µg/ml) for 5 days with and without anti-CCR4 antibody mogamulizumab (20µg/ml). Re-stimulation was performed on day 4 using HBs-specific peptides. In controls, no stimulation was given. Addition of golgi plug was done after 2 hours of incubation and staining was carried out according to the protocol explained in earlier experiments using CD3, CD4 and CD8 antibodies, followed by fixation and permeabilization of cells with FOXP3/transcription factor staining buffer set (eBioscience) and further staining with FOXP3, IL-10 and TGF-β1 antibodies. Samples were run on Cytek Aurora machine and data analysis was performed on FlowJo version 10 software.

***Multiplex Cytokine Bead Array Assay***

Levels of antiviral and anti-inflammatory/immunosuppressive cytokines including IFN-γ, TNF-α, IL-21, IL-4, IL-5, IL-10, IL-13, CCL17 and CCL22 were detected in the plasma of CHB patients and compared with the Vacc-HC by multiplex cytokine bead array assay using kits from Millipore company (Cat no. HCYTOMAG-60K and HCYP2MAG-62K) as per their instructions using Luminex technology.

***Statistical Analyses***

Chi-squared test, Mann-Whitney U test, unpaired t-test, and Wilcoxon matched-pairs signed rank-test were used to calculate the statistical significance. Correlation analysis was performed using Spearman’s rank correlation coefficient. All the analysis was performed on GraphPad Prism, version 9.0 software. Values have been presented as mean with standard deviation or median with range. P value < 0.05 was considered statistically significant and defined as follows: *p<0.05, **p<0.01, ***p<0.001 and ****p<0.0001.

**Table S1**

| S.N. | Antibody Name | Fluorochrome | Clone | Cat no. | Supplier |
| --- | --- | --- | --- | --- | --- |
| 1 | Live/Dead Zombie Aqua fixable viability stain | - | - | 423102 | Biolegend |
| 2 | CD3 | BV750 | SK7 | 344846 | BioLegend |
| 3 | CD4 | BV570 | RPA-T4 | 300534 | BioLegend |
| 4 | CD8 | PE/Dazzle | SK1 | 344744 | BioLegend |
| 5 | CCR4 | BV421 | L291H4 | 359413 | BioLegend |
| 6 | CCR4 | BV605 | L291H4 | 359418 | BioLegend |
| 7 | CCR6 | BV421 | G034E3 | 353408 | BioLegend |
| 8 | CCR7 | APC/Fire | G043H7 | 353246 | BioLegend |
| 9 | CCR10 | APC | 6588-5 | 341505 | BioLegend |
| 10 | CXCR3 | BV785 | G025H7 | 353738 | BioLegend |
| 11 | CXCR5 | Pacific Blue | J252D4 | 356918 | BioLegend |
| 12 | PD1 | AF700 | Eh12.2h7 | 329951 | BioLegend |
| 13 | CTLA4 | PE/Cyanine7 | BNI3 | 369614 | BioLegend |
| 14 | Tim3 | PE | F38-2E2 | 345006 | BioLegend |
| 15 | TIGIT | PerCP/Cynanine5.5 | A15153G | 372718 | BioLegend |
| 16 | 2B4 | FITC | C1.7 | 329506 | BioLegend |
| 17 | CD69 | BV650 | FN50 | 310934 | BioLegend |
| 18 | Ki67 | BV711 | Ki-67 | 350516 | BioLegend |
| 19 | CD45RO | PE/Cyanine5 | UCHL1 | 304208 | BioLegend |
| 20 | FOXP3 | FITC | 206D | 320106 | BioLegend |
| 21 | IFN-γ | BV421 | 4S.B3 | 502531 | BioLegend |
| 22 | TNF-α | PE/Cyanine7 | Mab11 | 502930 | BioLegend |
| 23 | IL-4 | PE/Cyanine7 | MP4-25D2 | 500824 | BioLegend |
| 24 | IL-5 | BV421 | TRFK5 | 504311 | BioLegend |
| 25 | IL-10 | APC | JES3-19F1 | 506807 | BioLegend |
| 26 | IL-13 | PerCP/Cynanine5.5 | JES10-5A2 | 501911 | BioLegend |
| 27 | IL-21 | APC | 3A3-N2 | 513008 | BioLegend |
| 28 | TGF-β1 | PE | TW4-2F8 | 349603 | BioLegend |
| 29 | CCL17 | PE | T48-854 | 566029 | BD Biosciences |
| 30 | CD107a | BV605 | H4A3 | 328634 | BioLegend |
| 31 | Perforin | FITC | B-D48 | 353309 | BioLegend |
| 32 | Granzyme B | PE/Cyanine7 | QA16A02 | 372213 | BioLegend |
|  |  |  |  |  |  |

**Fig. S1**

CD8

**Figure S1:** Hepatitis B core antigen (HBc) induces CCR4 expression in CHB patients. Line graph represents the expression of CCR4 on CD8 T cells in CHB patients after PBMC stimulation with HBc peptides for 5 days. PBMCs without any HBc stimulation were taken as controls.

**Fig. S2**

**Figure 2:** Blockade of CCR4 with Mogamulizumab, an anti-CCR4, boosts HBcAg-specific immune response in T cells of CHB patients. (A, B) Flow cytometry plot and line graphs designate the production of antiviral cytokines by CD4 and CD8 T cells in the presence and absence of anti-CCR4 antibody after 10 days of PBMC stimulation with HBc overlapping peptides along with IL-2.

**Fig. S3**

**Figure S3:** Blockade of CCR4 with Mogamulizumab inhibits CCL17 production by T cells. Line graph indicates the production of CCL17 upon HBc peptide stimulation for 10 days along with IL-2, in the presence and absence of anti-CCR4 antibody.
